# Patterns of Tree Species Diversity in Relation to Climatic Factors on the Sierra Madre Occidental, Mexico

**DOI:** 10.1371/journal.pone.0105034

**Published:** 2014-08-15

**Authors:** Ramón Silva-Flores, Gustavo Pérez-Verdín, Christian Wehenkel

**Affiliations:** 1 Universidad Juárez del Estado de Durango, Ciudad Universitaria, Durango, México; 2 Instituto Politécnico Nacional, CIIDIR Durango, Durango, México; 3 Instituto de Silvicultura e Industria de la Madera, Universidad Juárez del Estado de Durango, Ciudad Universitaria, Durango, México; Institute of Botany, Chinese Academy of Sciences, China

## Abstract

Biological diversity can be defined as variability among living organisms from all sources, including terrestrial organisms, marine and other aquatic ecosystems, and the ecological complexes which they are part of. This includes diversity within species, between species, and of ecosystems. Numerous diversity indices combine richness and evenness in a single expression, and several climate-based explanations have been proposed to explain broad-scale diversity patterns. However, climate-based water-energy dynamics appears to be an essential factor that determines patterns of diversity. The Mexican Sierra Madre Occidental occupies an area of about 29 million hectares and is located between the Neotropical and Holarctic ecozones. It shelters a high diversity of flora, including 24 different species of *Pinus* (ca. 22% on the whole), 54 species of *Quercus* (ca. 9–14%), 7 species of *Arbutus* (ca. 50%) and many other trees species. The objectives of this study were to model how tree species diversity is related to climatic and geographic factors and stand density and to test the Metabolic Theory, Productivity-Diversity Hypothesis, Physiological Tolerance Hypothesis, Mid-Domain Effect, and the Water-Energy Dynamic Theory on the Sierra Madre Occidental, Durango. The results supported the Productivity-Diversity Hypothesis, Physiological Tolerance Hypothesis and Water-Energy Dynamic Theory, but not the Mid-Domain Effect or Metabolic Theory. The annual aridity index was the variable most closely related to the diversity indices analyzed. Contemporary climate was found to have moderate to strong effects on the minimum, median and maximum tree species diversity. Because water-energy dynamics provided a satisfactory explanation for the patterns of minimum, median and maximum diversity, an understanding of this factor is critical to future biodiversity research. Quantile regression of the data showed that the three diversity parameters of tree species are generally higher in cold, humid temperate climates than in dry, hot climates.

## Introduction

Biological diversity can be defined as variability among living organisms from all sources, including terrestrial organisms, marine and other aquatic ecosystems, and the ecological complexes which they are part of; this includes diversity within species, between species, and of ecosystems” [1]. McNeely [2] considered biodiversity as an umbrella term for the degree of variety in nature, including the number and frequency of ecosystems, species or genes in a given assemblage. Biodiversity is usually considered at three different levels: “genetic diversity”, “species diversity” and “ecosystem diversity” [3], [4]. Biodiversity is not simply the number of different genes, species, ecosystems, or any other group of things in a defined area. The composition, structure and function determine and also constitute the biodiversity of an area [5].

Various diversity indices have been established, but very few are commonly applied in ecological studies, e.g. richness [6], the Shannon index [7], Simpson index [8]. However, many of these measures can be converted into members of a family of explicit diversity indices, also known as Hill family [9], [10] or Rényi-diversity [11], [12], [13], [14].

Climate is a key factor that determines the distribution of plant species [15]. Global climate change is an enormous challenge to those responsible for developing conservation strategies for forest species [16] because it can modify the distribution of genes and species as well as the composition of vegetation and also create new biogeoclimatic zones with individual species [17], [18]. The amount of predicted decoupling between biomes and their suitable climatic habitat will vary greatly between geographic areas and will depend on the level of greenhouse gas emissions in this century [19]. Wright et al., [20] and Hawkins et al., [21] have suggested that one of the most important patterns in ecology is the variation in broad-scale variation in taxonomic richness with climate and geography.

Several climate-based explanations have been proposed to explain broad-scale diversity patterns, e.g., the Mid-Domain Effect [22], Productivity-Diversity hypothesis (the more individuals hypothesis), the Physiological Tolerance Hypothesis, the Speciation Rates Hypothesis and Species-Temperature Hypotheses such as the Metabolic Theory [23], [24]. However, climate-based water-energy dynamics also appears to be an essential factor that determines patterns of diversity [25]. According to this theory, the influence of water decreases and the influence of energy increases with absolute latitude [26].

Recent studies have demonstrated a strong relationship between total species richness and temperature, precipitation, and net primary productivity [21], [23], e.g., in South Africa [24], China [27], [28], [29], Ecuador, Costa Rica, Mexico and Tanzania [30], USA and Canada [29], India [31], [32], and Europe [33]. In a meta-analysis of 46 broad-scale data sets of species richness for a wide range of terrestrial plant, invertebrate, and ectothermic vertebrate groups throughout the world, the authors found that the relationship between richness and temperature is both taxonomically and geographically conditional [34]. However, there is no evidence of a universal response of diversity to temperature. For strictly tropical taxa such as palms [26], it has been confirmed that the influence of water and energy on species richness varies across large climatic gradients spanning tropical to temperate and arctic zones and also within megathermal climates. In a study carried out in the Amazon rainforest, Steege et al., found that dry season length, while only weakly correlated with average tree *α*-diversity, is a strong predictor of tree density and of maximum tree *α*-diversity [35]. Denser forests are more diverse than sparser forests, even when a diversity measure is used to correct for sample size [36].

The Mexican Sierra Madre Occidental occupies an area of about 29 million hectares and is located between the Neotropical and Holarctic ecozones. It shelters a high diversity of flora and fauna [37], [38] including 24 different species of *Pinus* (ca. 22% on the whole), 54 species of *Quercus* (ca. 9–14%), 7 species of *Arbutus* (ca. 50%) and many other trees species [39], [40]. Although this ecosystem is the largest forest biomass reserve in the country, little is known about the diversity of tree species [41].

Therefore, the objective of this study was to model how tree species diversity is related to climatic factors and stand density in the Sierra Madre Occidental and to test the Metabolic Theory, Productivity-Diversity Hypothesis, Physiological Tolerance Hypothesis [23], [24], Mid-Domain Effect [22], and the Water-Energy Dynamic Theory [25], [26]. The results may serve as tool for evaluating tree species diversity on the basis of climatic variables.

## Material and Methods

The CONAFOR (National Forestry Commission), Mexico (http://www.conafor.gob.mx/portal/) provides the data set. No specific permissions were required for these locations/activities. We confirm that the field studies did not involve endangered or protected species and provide the specific location of your study (e.g. GPS coordinates). There no were vertebrate studies.

### Study area

**Figure 1:**
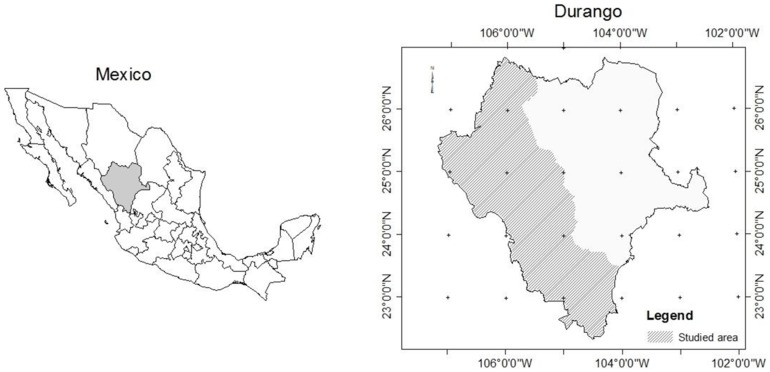
Location of study area.

**Figure 2:**
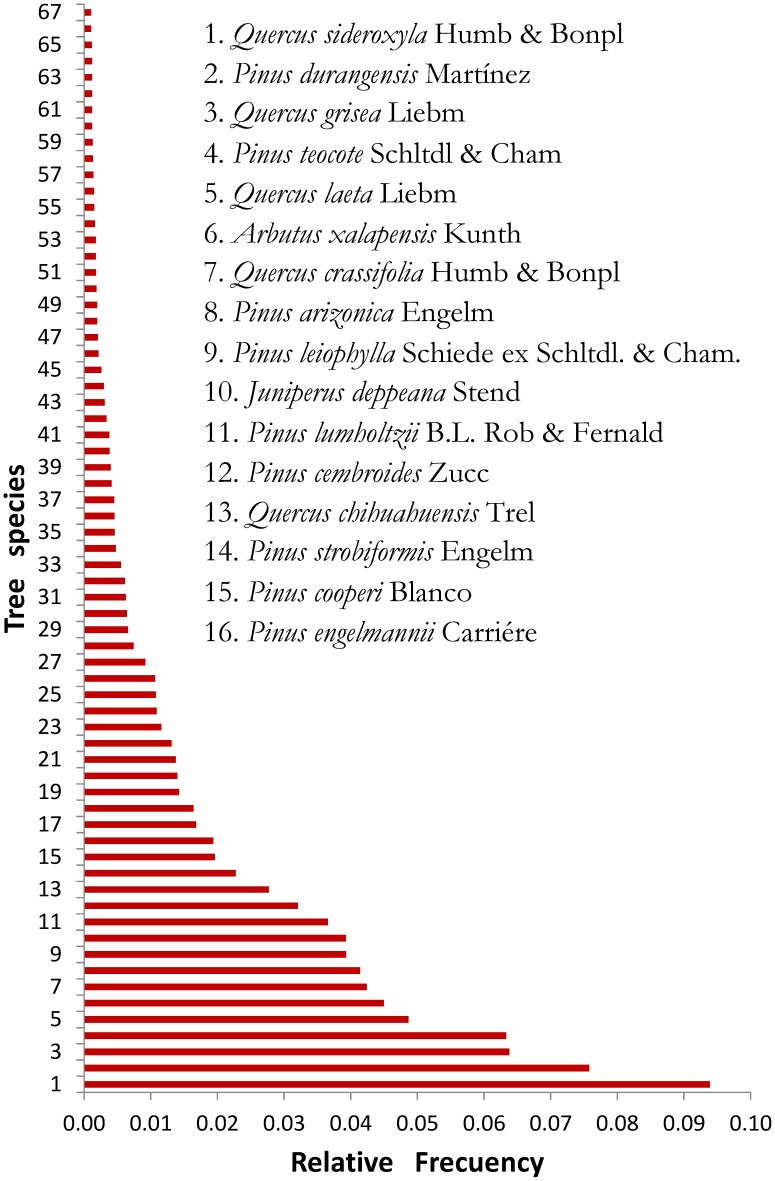
Relative frequency of trees species in the Sierra Madre Occidental. The names of the 16 most frequent species found in the study area are shown.

The study was conducted in the State of Durango (22°20′49″ N - 26°46′33″ N; 103°46′38″ W -107°11′36″ W), which occupies about 23% of the Sierra Madre Occidental ecosystem (Figure 1). The area covers a surface of approximately 6.33 million ha. The elevation above sea level varies between 363 and 3,190 mm (average 2,264 m). The climate ranges from temperate to tropical, with a total annual rainfall varying from 443 to 1,452 mm and an annual average of 917 mm. The mean annual temperature varies from 8.2 to 26.2°C, and the annual average is 13.3°C [42], [43]. The predominant forest types are uneven-aged pine-oak, often mixed with *Pseudotsuga menziesii*, *Arbutus* spp., *Juniperus* spp. and other tree species. The relative frequency of 67 tree species out of the 327 existing in the 1,632 sample plots is shown in Figure 2. These 67 trees species represent 97% of the cumulative relative frequency.

About 2 million ha of the forest land is mainly managed by selective removal, with only a small amount of other harvesting methods (less than 5% of the productive forest area). Clear felling is almost unknown, except in some parts of central and northern Durango [39]. The forest structure and density are due to the particular climatic and soil conditions and also to the specific management practices, which include use of the forest as pasture. Cattle grazing generate a large amount of the forest owners' income, which requires fairly open forest conditions [44].

### Sampling sites

**Figure 3:**
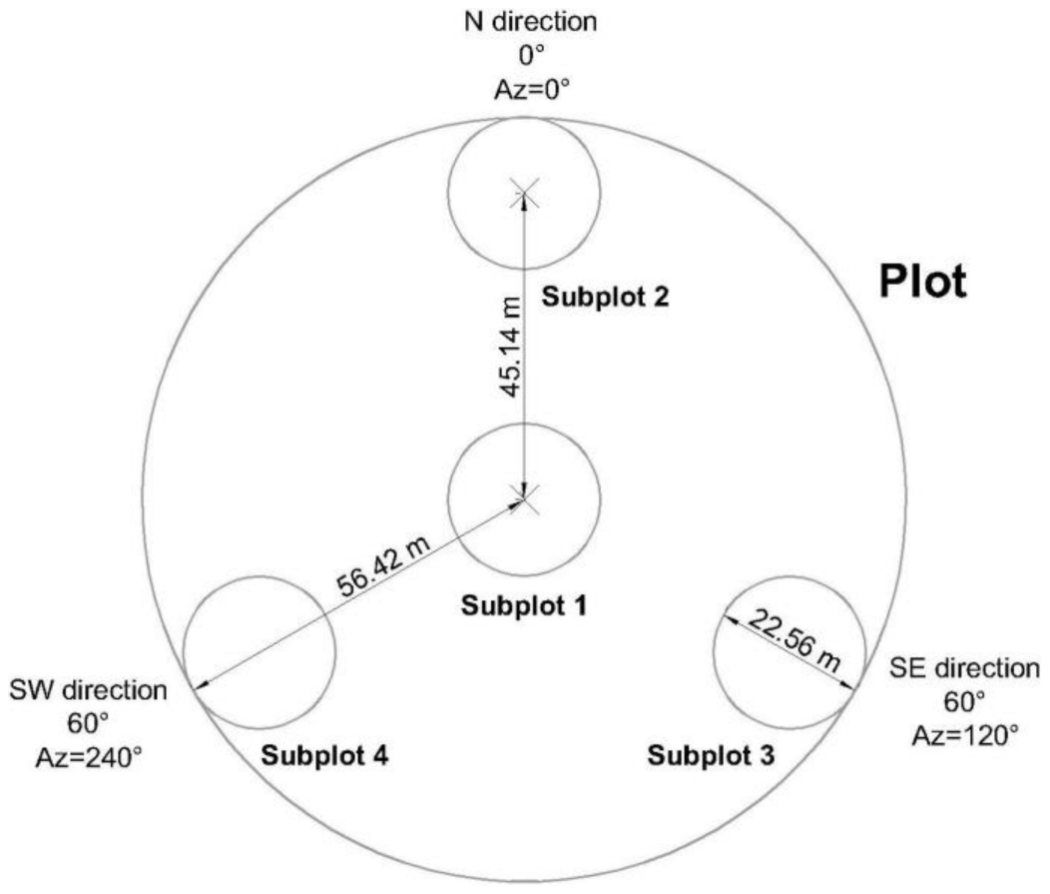
Design of a single plot included in the National Forest and Soil Inventory (modified from CONAFOR, 2004). The plot is composed of four 400 m^2^ subplots distributed as an inverse ‘Y’. Abbreviations: Az = Azimuth, m = meters, N = North; SE = Southeast; SW = Southwest

The vegetation data were obtained from the National Forestry Commission, which is responsible for implementing the National Forest and Soil Inventory in Mexico. The sampling design included approximately 25,000 plots [43], distributed throughout the forest area in 5 by 5 km grids (Figure 3). Of the 1,737 plots located in the State of Durango, 1,632 were used in this study. A plot (1,600 m^2^) consists of four 400 m^2^-circular subplots, as shown in Figure 3 [45]. In each plot, the total number of trees, genera and species were recorded along with altitude, latitude and longitude. Tree data were taken from individuals with at least 3 m tall and diameter at breast height (1.30 m) of 7.5 cm.

### Climate model

The climate model of Rehfeldt [45], [46], [47], which is based on the thin plate splines of Hutchinson [42], [47] and [48] was used to estimate climate variables in each plot. The model produces climate surfaces from normalized monthly values of total precipitation and mean, maximum and minimum temperatures collected between 1961 and 1990 from approximately 6,000 weather stations (183 stations in Durango State). Hutchinson's software was used to predict the following: (i) the climate at specific points, identified by latitude, longitude and elevation, and (ii) the climate along gridded surfaces. Point estimates were obtained using a national database run by the University of Idaho (http://forest.moscowfsl.wsu.edu/climate/) which requires point coordinates (latitude, longitude, and elevation) as the main inputs (see [45] and [46] for technical procedures). Sixteen variables were derived from the original data, which also included an annual aridity index (AAI), defined as the ratio of square root of degree days >5°C (DD5) to mean annual precipitation (MAP). Higher values of the aridity index indicate more arid climate, whereas smaller values indicate either very warm and very humid or cold and humid climates. The AAI index is a powerful climatic variable for describing and predicting distributions of pine species [47]. Other variables are described in Table 1.

**Table 1:** Basic statistics of the geographical, climatic and species diversity variables studied.

Variable	Acronym	Units	Means	Std. Dev.	Min	Max
Longitude	LONG	Deg	−105.6	0.690	−107.2	−104.1
Latitude	LAT	Deg	24.5	1.066	22.4	26.8
Elevation above sea level	ELEV	m	2,239.0	506.1	390.1	3,156.0
Mean annual temperature	MAT	°C	13.5	3.8	8.3	26.2
Mean annual precipitation	MAP	mm	916.8	218.7	444.6	1,450.0
Growing season precipitation, April-September	GSP	mm	694.3	143.3	378.0	1,033.0
Mean temperature in the coldest month	MTCM	°C	8.3	4.0	2.9	21.4
Minimum temperature in the coldest month	MMIN	°C	−0.867	4.6	−6.8	12.0
Mean temperature in the warmest month	MTWM	°C	18.1	3.8	12.6	30.5
Maximum temperature in the warmest month	MMAX	°C	26.5	3.4	20.6	40.2
Julian date of the last freezing date of spring	SDAY	Days	112	49	1	182
Julian date of the first freezing date of autumn	FDAY	Days	303	29	251	365
Length of the frost-free period	FFP	Days	192	76	79	365
Degree days >5°C	DD5	Days	3,200	1,317	2,725	7,640
Degree days >5°C accumulating within the frost-free period	GSDD5	Days	2,373	1,537	1,780	7,551
Julian date when the sum of degree days >5°C reaches 100	D100	Days	36	19	36	84
Degree days <0°C	DD0	Days	8	11	1	65
Minimum degree days <0°C	MMINDD0	Days	503	367	484	1,323
Annual aridity index (the ratio of square root of DD5 to MAP)	AAI	Days^0.5^*mm^−1^	0.064	0.020	0.060	0.154
Number of individuals per plot	NIP	Trees	70	43	62	348
Species richness index per plot	*v_0_*		6.5	2.9	6	17
Effective species number per plot	*v_2_*		3.5	1.6	3.3	9.7
Amount of prevalent tree species	*v_∞_*		2.42	0.98	2.30	6.92

### Calculation of diversity

The species diversities (Table 1) were calculated by the so-called Hill numbers, Hilĺs family, or diversity profile *v_a_* [9], [10], where *a* is a real number ranging from zero to infinity. The general concept underlying classification with *v_a_* is that with increasing *a*, the most frequent types of species increasingly determine the diversity of a collection to a greater degree than the less frequent types and that the extent to which this is true increases with increasing values of parameter *a*. Among the most desirable characteristics of a measure of diversity is that *v_a_* satisfies the following requirements, irrespective of the value of *a*: (i) for a given number of variants it assumes its largest value exactly when all these variants are equally frequent, and this value equals the number of tree species, (ii) it increases as two variants approach equal frequencies, and (iii) it increases when one variant is subdivided into several varieties.

Considered as a function of *a*, *v_a_* describes a diversity profile for each frequency distribution. The following are the most illustrative values of the subscript *a* in such diversity profiles: (i) *a* = 0, where the diversity is equivalent to the total number of variants; (ii) *a* = 2 as the effective number used in most genetic studies, and (iii) *a* = ∞, where only the relative frequency of the most frequent variant determines the diversity. In the present study, the diversity profiles are represented by all three diversities for each sample plot. Thus, each population was characterized by the *total* number, the *effective* number [49], inherent in Simpson diversity (*D* = ∑p_i_^2^) [8], and the amount of *prevalent* variants [10]. All variants have the same abundance when *v_0_*, *v_2_* and *v*_∞_ have the same value.

Formally (*p_i_* = relative frequency of a tree species *i*):
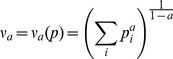
(1)


### Data analysis

Plots without trees were excluded. IBM SPSS Statistics and SAS/STAT software [50] were used to calculate descriptive statistics, and to carry a Principal Component Analysis (PCA) and Spearman's Correlation Analysis.

Principal Component Analysis (PCA) with varimax rotation [51], [52] was used to reduce the number of variables into underlying factors (components) of climatic and geographic variables. Two factors were clearly identified and accounted for 85% of the total variance. Bartlett's test of sphericity, which evaluates the hypothesis that the correlations in the correlation matrix are zero, was equal to 0.864 (p<0.001), i.e. it was highly significant. The factor loadings indicated that the main factor (factor 1) included MAT, MTCM, FDAY, DD5, FFP, GSDD5, SDAY, MMIN, D100, MTWM, MMINDD0, ELEV, MMAX and DD0. This factor was identified as the temperature group. The second factor only included GSP, MAP, LAT, and AAI and was designated as the precipitation group. The 14 variables included in the temperature group represented 69% of the total variance of the diversity of tree species in the Sierra Madre Occidental, while the precipitation group comprised four variables representing 16% of the total variance.

SAS/STAT software [50] was also used to calculate the well-known Spearman's coefficient (*r_s_*) to determine how diversity is correlated with climatic and geographic variables and the number of individuals per plot (NIP).

In addition, R software, version 2.13.1 [53] and the ‘quantreg’ module were used to construct quantile models for tree species diversity with climatic and geographic variables that were included in the PCA main factor (factor 1), and for which the absolute values of *r_s_* were larger than 0.3 (Table 2). Additionally, quantile models for tree species diversity with NIP and AAI were generated because of a strong correlation (*r_s_*) between these variables and tree species diversity (Table 2). This method was also used for nonlinear regression because the variables were analyzed without prior transformation.

**Table 2:** Matrix of Spearman correlation (r_S_) between climate variables.

VARIABLES	LONG	LAT	ELEV	MAT	MAP	GSP	MTCM	MMIN	MTWM	MMAX	SDAY	FDAY	FFP	DD5	GSDD5	D100	DD0	MMINDD0	AAI
LAT	−0.786																		
ELEV	**0.022^ns^**	0.206																	
MAT	0.183	−0.296	−0.902																
MAP	−0.271	−0.331	−0.153	−**0.017^ns^**															
GSP	−0.137	−0.458	−0.194	0.066	0.982														
MTCM	0.230	−0.405	−0.886	0.981	0.101	0.188													
MMIN	0.212	−0.432	−0.831	0.938	0.193	0.275	0.980												
MTWM	0.117	−0.182	−0.905	0.983	−0.096	−0.026	0.935	0.873											
MMAX	**0.054^ns^**	−**0.035^ns^**	−0.823	0.895	−0.237	−0.179	0.813	0.718	0.950										
SDAY	−0.239	0.372	0.835	−0.967	−**0.041^ns^**	−0.132	−0.973	−0.965	−0.924	−0.789									
FDAY	0.200	−0.411	−0.894	0.965	0.160	0.241	0.985	0.977	0.918	0.777	−0.972								
FFP	0.214	−0.398	−0.883	0.971	0.119	0.203	0.984	0.975	0.926	0.786	−0.985	0.996							
DD5	0.178	−0.289	−0.904	0.999	−**0.022^ns^**	**0.059^ns^**	0.978	0.934	0.986	0.901	−0.965	0.962	0.968						
GSDD5	0.188	−0.319	−0.895	0.995	**0.020^ns^**	0.103	0.984	0.955	0.971	0.864	−0.982	0.977	0.984	0.994					
D100	−0.225	0.385	0.877	−0.984	−0.069	−0.158	−0.997	−0.976	−0.939	−0.818	0.979	−0.981	−0.984	−0.982	−0.986				
DD0	−0.296	0.364	0.807	−0.931	**0.031^ns^**	−0.057	−0.948	−0.926	−0.884	−0.753	0.926	−0.935	−0.937	−0.928	−0.933	0.948			
MMINDD0	−0.224	0.402	0.837	−0.960	−0.117	−0.205	−0.984	−0.991	−0.903	−0.757	0.987	−0.981	−0.986	−0.957	−0.975	0.987	0.935		
AAI	0.271	0.087	−0.451	0.647	−0.741	−0.674	0.538	0.446	0.708	0.778	−0.584	0.479	0.517	0.652	0.617	−0.566	−0.568	−0.520	
NIP	−0.251	0.238	0.399*	−0.467*	0.108	**0.046^ns^**	−0.447*	−0.407*	−0.463*	−0.426*	0.458*	−0.434*	−0.446*	−0.467*	−0.458*	0.453*	0.422*	0.437*	−0.381*

Notes: ns  =  no significant correlations (p>0.01), *  = Association of variables with significant correlation (Spearman coefficient >|0.3| and p<0.01).

Quantile regression is useful for analysis of non-normally distributed data sets. This nonparametric method is used to examine specific segments of the conditional distribution and upper or lower quantile function of several covariates of interest. The technique is based on minimizing the absolute error and estimated functions for the median (as a robust version of the mean) and other quantiles conditional for a random variable with distribution function *F*(y) = Prob (*Y*≤y). The quantile (*τ*) is defined by the inverse *Q* (*τ*) = inf{y: *F*(y)≥*τ*} where 0<*τ* <1. The median equals *Q* (0.5) [54], [55]. The quantile regression procedure computes the quantile function *Q* (*τ* | *X = x*).

In the nonlinear quantile regressions, parameters *a*, *b*, *c*, *d* and *e* were used with the form *y = dx+e* to *y = ax^4^+bx^3^+cx^2^+dx+e* to analyze the polynomial family of univariate models from 2 to 5. After preselection of various linear and nonlinear multivariate models, the linear function *v_a_ = aMAP+bMMAX+c* was used to test the water energy dynamics theory (WED). The mean annual precipitation (MAP) and the maximum temperature in the warmest month (MMAX) were selected for the following reasons: i) MAP is most closely correlated with *v_a_*, in the set of precipitation variables, and MMAX, in the group of temperature variables with *v_a_* (Table 3) calculated by stepwise multiple regression, and ii) these two variables display a very low degree of collinearity (Table 2).

**Table 3:** Matrix of Spearman correlation (r_S_) calculated from the climate and diversity variables.

VARIABLES	LONG	LAT	ELEV	MAT	MAP	GSP	MTCM	MMIN	MTWM	MMAX	SDAY	FDAY	FFP	DD5	GSDD5	D100	DD0	MMINDD0	AAI	NIP
*v_0_*	−0.127	**0.021^ns^**	0.272	−0.317*	0.248	0.213	−0.276	−0.228	−0.338*	−0.358*	0.295	−0.258	−0.271	−0.318*	−0.302*	0.288	0.265	0.265	−0.412*	0.472*
*v_2_*	−0.082	−**0.003^ns^**	0.237	−0.266	0.206	0.178	−0.232	−0.188	−0.287	−0.310*	0.244	−0.216	−0.225	−0.267	−0.253	0.241	0.220	0.219	−0.345*	0.267
*v_∞_*	−0.069	−**0.007^ns^**	0.230	−0.256	0.187	0.161	−0.225	−0.186	−0.275	−0.296	0.237	−0.211	−0.218	−0.257	−0.245	0.234	0.213	0.214	−0.323*	0.220

Notes: ns =  no significant correlations (*p*>0.01),*  = Association of variables with significant correlation (Spearman coefficient>|0.3| and p<0.01).

The values of the quantiles (*τ*) were set at 0.9 for the upper limit, 0.5 for the median, and 0.1 for the lower limit. The quantile 0.9 was used to estimate the trend of maximum diversity with climate variables and the quantile 0.1 for minimum diversity with climate variables and NIP. The maximum and minimum diversity should serve as additional diversity measures. The linear and nonlinear model functions (*object<- lm* and *object<-nlrq*) in *R* [53] and the correlation (*r*) between the predicted and observed *v_a_* were used to compute the coefficient of determination (*r^2^*) of the bi- and multivariate quantile and non-quantile models (R-code for (pseudo) *r^2^* for quantile regression: *res<-residuals(object, type = “rho”) R2<- (cor(predict.nlrq(object), res + predict.nlrq(object)))∧2*).

## Results

Tree species richness (*ν_0_*) was almost twice as high as effective tree diversity (*ν_2_*) and *ν_2_* was almost one and a half times greater than the number of prevalent tree species (*ν_∞_*), i.e. the abundance of the tree species was different (Table 1, Figure 2). The matrix of Spearman coefficients (*r_s_*) revealed significantly weak to moderate correlations between climatic variables and also between climatic variables and diversity indices (Table 3). Climate had a greater effect on *ν_0_* than on *ν_2_* and *ν_∞_*, i.e., the climate had a greater influence in determining the number of rare tree species (which is the main component of the species richness index) than in determining the number of more frequent species.

**Figure 4:**
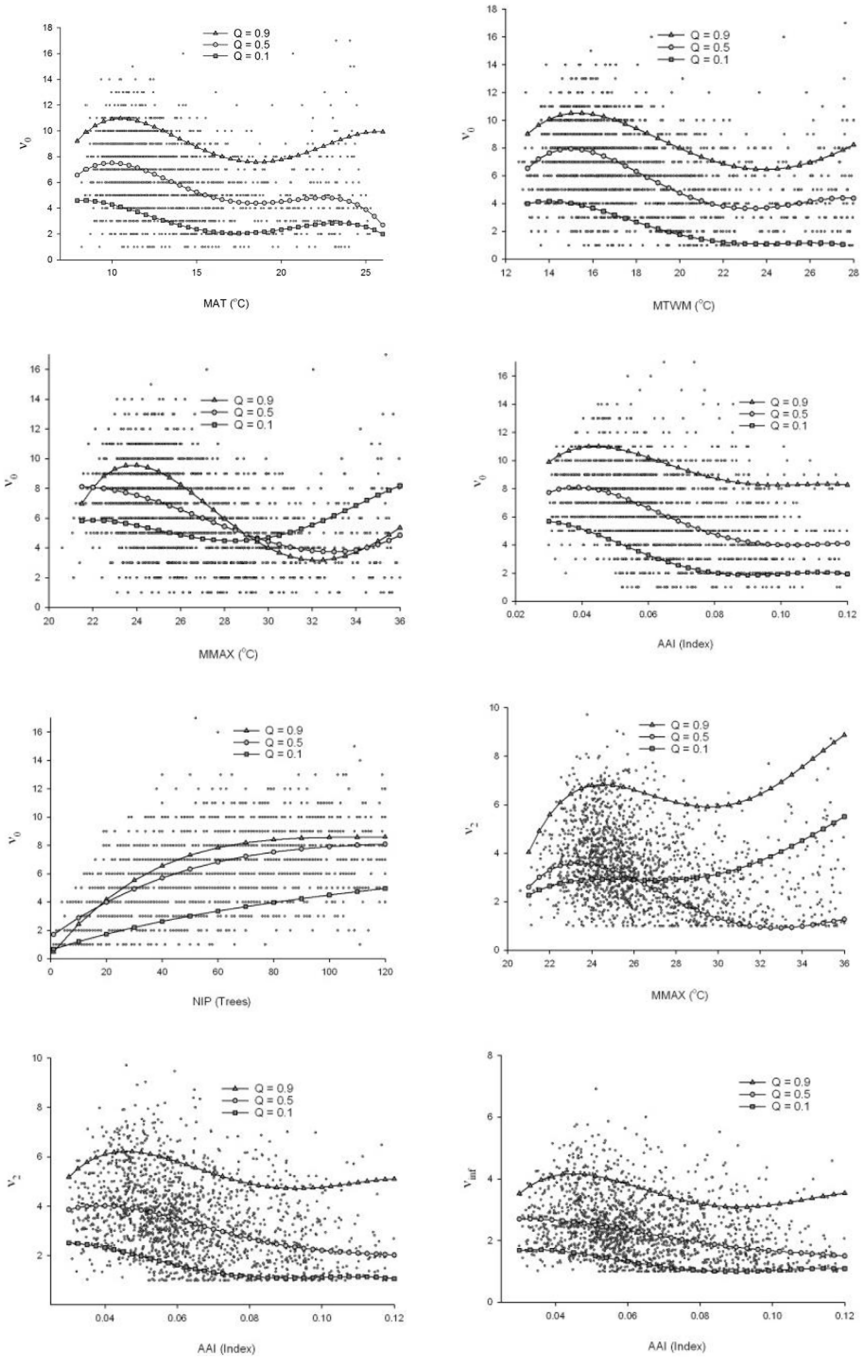
Series of curves of the quantile regression for tree species richness (*v_0_*), the effective tree species number (*v_2_*) and the amount of prevalent tree species (*v_∞_*) for quantile levels of (*τ*) 0.1, 0.5, and 0.9. The climatic variables are as follows: mean annual temperature (MAT), mean temperature in the warmest month (MTWM), maximum temperature in the warmest month (MMAX), annual aridity index (AAI), and finally, the number of individuals per plot (NIP).

The most significant relationships were the annual aridity index (AAI), maximum temperature in the warmest month (MMAX), the number of individuals per plot (NIP) and the tree species diversity (*ν_a_*), with *r_s_* values ranging from −0.32 to −0.41, from −0.31 to −0.36, and 0.22 to 0.47, respectively. The temperature variables mean annual temperature (MAT), mean temperature in the warmest month (MTWM), degree days >5°C (DD5) and degree days >5°C accumulated within the frost-free period (GSDD5) were also highly significantly and negatively correlated with *ν_a_* (Table 3). The three linear multivariate non-quantile models of *v_a_* = function (MAP, MMAX) showed weak coefficients of determination (*r^2^* = 0.08 (*v_∞_*), 0.09 (*v_2_*) and 0.13 (*v_0_*)). However, the nine non-linear multivariate quantile models of *ν_a_* with MAP and MMAX exhibited moderate to high coefficient values (*r^2^* = 0.55–0.71 (*v_∞_*), 0.58–0.75 (*v_2_*) and 0.50–0.83 (*v_0_*)) (Table 6). In addition, the non-linear bivariate quantile models of *ν_a_* with climatic factors as well as stand density presented moderate to high coefficient values (*r^2^* = 0.31–0.79 (*v_∞_*), 0.36–0.81 (*v_2_*) and 0.36–0.90 (*v_0_*) (Table 4, Figure 4). Plots with minimal tree diversity were often located between 1,800 and 2,200 m above sea level (at AAI≈0.120–0.154 index value) at the border of the Chihuahuan desert, facing inland.

**Table 4:** The parameters and probability of error (p) of polynomial nonlinear quantile regression models of diversity indices with climatic variables at the 0.9, 0.5 and 0.1 quantile levels (τ).

Variables	*τ*	*r^2^*	Parameters	a	b	c	d	e
*v_0_* – MAT	0.9	0.68	value	−0.0009	0.0628	−1.6188	17.2090	−53.4731
			*p*	0.1676	0.1142	0.0807	0.0647	0.1159
			error	0.0006	0.0397	0.9261	9.3097	33.9910
	0.5	0.36	value	−0.0009	0.0618	−1.5001	15.0798	−45.9928
			*p*	0.0070	0.0048	0.0037	0.0040	0.0163
			error	0.0003	0.0219	0.5162	5.2265	19.1328
	0.1	0.76	value	−0.0005	0.0308	−0.7001	6.3320	−15.0739
			*p*	0.2092	0.2280	0.2680	0.3494	0.5672
			error	0.0004	0.0255	0.6318	6.7644	26.3377
*v_0_* – MTWM	0.9	0.70	value	−0.0007	0.0678	−2.3462	34.2400	−168.4842
			*p*	0.2653	0.1872	0.1324	0.0978	0.0957
			error	0.0006	0.0514	1.5583	20.6660	101.0643
	0.5	0.46	value	−**0.0013**	**0.1176**	−**3.7609**	**51.5186**	−**247.7348**
			*p*	0.0006	0.0003	0.0002	0.0001	0.0001
			error	0.0004	0.0324	0.9900	13.1527	64.0229
	0.1	0.79	value	−0.0007	0.0587	−1.8170	23.9195	−109.1866
			*p*	0.0412	0.0412	0.0455	0.0572	0.0895
			error	0.0003	0.0287	0.9077	12.5663	64.2657
*v_0_* – MMAX	0.9	0.80	value	−**0.0014**	**0.1764**	−**8.2776**	**168.7058**	−**1252.0169**
			*p*	0.0069	0.0025	0.0009	0.0003	0.0001
			error	0.0005	0.0582	2.4830	46.6776	326.5024
	0.5	0.43	value		0.0057	−0.4649	12.0452	−92.7230
			*p*		0.0003	0.0006	0.0020	0.0121
			error		0.0501	2.1334	40.0340	279.2817
	0.1	0.81	value	−0.0006	0.0753	−3.2486	60.8045	−411.7345
			*p*	0.0986	0.0907	0.0869	0.0886	0.0998
			error	0.0004	0.0445	1.8964	35.6846	250.0008
*v_0_* – AAI	0.9	0.66	value	−**571021.2704**	**195722.2198**	−**23593.7069**	**1132.4081**	−**7.6657**
			*p*	0.0000	0.0000	0.0000	0.0000	0.0134
			error	187.8099	8876.5940	2096.1069	150.2528	3.0954
	0.5	0.48	value	−**453491.3979**	**158284.4650**	−**19007.7266**	**860.1545**	−**4.8716**
			*p*	0.0000	0.0000	0.0000	0.0000	0.0008
			error	572.1734	3589.5689	863.7811	63.9525	1.4532
	0.1	0.85	value	−**426868.9341**	**131061.4899**	−**13434.8946**	**476.2212**	**0.2906**
			*p*	0.0000	0.0000	0.0000	0.0004	0.9238
			error	1530.9391	7867.5688	1840.7669	134.5190	3.0364
*v_0_* – NIP*	0.9	0.90	value	−**1.7209E-08**	**1.1462E-05**	−**2.5805E-03**	**0.24392**	**2.67529**
			*p*	0.00622	0.00016	0.00000	0.00000	0.00000
			error	0.00000	0.00000	0.00047	0.02793	0.41146
	0.5	0.61	value	−4.7616E-09	3.8079E-06	−1.1208E-03	0.14230	1.56533
			*p*	0.20723	0.03372	0.00007	0.00000	0.00000
			error	0.00000	0.00000	0.00028	0.01590	0.21781
	0.1	0.87	value	−2.4871E-09	1.5035E-06	−0.00037	0.06309	0.60922
			*p*	0.52797	0.41469	0.15491	0.00000	0.00014
			error	0.00000	0.00000	0.00026	0.01239	0.15939
*v_2_* – MMAX	0.9	0.67	value	−**0.0010**	**0.1180**	−**5.3449**	**105.9564**	−**769.6654**
			*p*	0.0000	0.0000	0.0000	0.0000	0.0000
			error	0.0002	0.0242	1.0382	10.6058	137.2664
	0.5	0.37	value	−**0.0005**	**0.0627**	−**2.8916**	**58.0009**	−**423.3663**
			*p*	0.0009	0.0004	0.0002	0.0001	0.0001
			error	0.0002	0.0176	0.7624	14.5742	103.5761
	0.1	0.75	value	−0.0003	0.0355	−1.5887	30.9829	−220.1202
			*p*	0.0428	0.0357	0.0311	0.0290	0.0305
			error	0.0001	0.0169	0.7364	14.1736	101.6441
*v_2_* – AAI	0.9	0.64	value	−**413660.0818**	**143466.7997**	−**17458.6761**	**855.7508**	−**8.3143**
			*p*	0.0000	0.0000	0.0000	0.0000	0.0000
			error	1451.8510	4644.0345	1119.7915	84.8163	1.9990
	0.5	0.36	value	−149515.0681	53535.0264	−6662.4690	310.2461	−0.7709
			*p*	0.0000	0.0000	0.0000	0.0000	0.4506
			error	851.3523	2033.2694	500.2416	40.0305	1.0217
	0.1	0.81	value	−172936.4467	52569.6039	−5359.5093	191.1053	0.3302
			*p*	0.0000	0.0000	0.0000	0.0064	0.8312
			error	1565.8892	4205.3104	978.0459	70.0480	1.5484
*v_∞_* – AAI	0.9	0.65	value	−**292007.5163**	**101441.4217**	−**12274.8255**	**594.2765**	−**5.7624**
			*p*	0.0000	0.0000	0.0000	0.0000	0.0000
			error	0.0000	2923.4180	651.1020	46.4309	1.0637
	0.5	0.31	value	−**50472.4434**	**17865.9822**	−**2180.6198**	**91.9919**	**1.4579**
			*p*	0.0000	0.0000	0.0000	0.0000	0.0015
			error	486.8971	942.9610	234.4576	18.5449	0.4577
	0.1	0.79	value	−115122.9857	36829.1202	−4014.9091	163.7877	−0.5152
			*p*	0.0000	0.0000	0.0000	0.0000	0.3975
			error	247.6810	1317.2320	316.3406	24.4349	0.6087

Notes: * The parameters obtained in the models are valid for a range in the number of trees per plot from 1 to 120. The statistically significant parameters in the models (*p*<0.05) are indicated by bold numbers.

Analysis of ten non-linear quantile (*τ*) regressions of the three ranges of tree species diversity (*v_0_*, *v_2_* and *v_∞_*) and six climate variables as well as the number of individuals per plot (NIP) were carried out for absolute *r_s_* values >0.30 between the three tree species diversities and climate variables and NIP (Tables 2 and 3). The results showed five significant fits for regression of *v_0_* and *v_2_* with MMAX for the maximum, median, and minimum diversity of tree species (quantile levels (*τ*) = 0.1, 0.5 and 0.9). The regression of tree species diversity with AAI and NIP resulted in five good-fit models (Table 4). In contrast, DD5 and GSDD5 were not significantly correlated with the three diversity indices according to the polynomial models. Quantile regressions of species richness (*v_0_*) with statistically significant parameters such as *a*, *b*, *c*, *d*, and *e* were mainly found for MAT, MTWM, MMAX, AAI and NIP. Quantile regressions of the effective species number (*v_2_*) with statistically significant parameters were only observed for MMAX and AAI. The number of prevalent tree species (*v_∞_*) only yielded a significant quantile regression with AAI (Table 4, Figure 4).

All temperature-diversity-curves were hollow-shaped (with a local minimum) for the median (*τ* = 0.5) and maximum diversity (*τ* = 0.9). Tree species diversity is generally higher in colder temperate climates and in hot climates, but tends to fall to a local minimum in milder climates. The local maxima of the tree species richness and effective diversity (*ν_0_* and *ν_2_* at *τ* = 0.9) were found in temperate climate areas. In some cases, the curves were sinuous, with a local maximum and local minimum of tree species diversity. However, the relationship between AAI and the diversity variables was essentially linear or constrained within a linear limit. Finally, the NIP-tree diversity curves formed saturation curves (Figure 4, Table 5).

**Table 5:** Points of local minimum (Min) and maximum values (Max) of the tree species richness (*v_0_*), the effective tree species number (*v_2_*) and the number of prevalent tree species (*v_∞_*) with (*τ*) 0.5, and 0.9 as the quantile levels.

Diversity index	Climatic variable	Unit	*τ* = 0.5		*τ* = 0.9	
			Min	Max	Min	Max
*v_0_*	MAT	°C	(18.48; 4.39)	(9.96; 7.51)	(18.57; 7.57)	(10.48; 10.96)
	MTWM	°C	(23.30; 3.65)	(14.90; 7.94)	(23.90; 6.46)	(15.31; 10.52)
	MMAX	°C	(32.92; 3.73)	(21.35; 8.12)	(32.20; 3.17)	(23.80; 9.58)
	AAI	Days^0.5^/mm	(0.10; 3.99)	(0.04; 8.11)	(0.10; 8.26)	(0.04; 11.01)
*v_2_*	MMAX	°C	(32.75; ≈1.00)	(23.50; 3.60)	(29.50; 5.91)	(24.50; 6.83)
	AAI	Days^0.5^/mm	–	(0.04; 4.02)	(0.09; 4.72)	(0.05; 6.22)
*v_∞_*	AAI	Days^0.5^/mm	–	(0.03; 2.70)	(0.09; 3.09)	(0.04; 4.17)

The climatic variables are as follows: mean annual temperature (MAT), mean temperature in the warmest month (MTWM), maximum temperature in the warmest month (MMAX), and the annual aridity index (AAI).

**Table 6:** The parameters a, b and c, probability of error (*p*) and the error of linear multivariate models of diversity indices (error) with climatic variables MMAX and MAP at the 0.1, 0.5 and 0.9 quantile levels (*τ*).

Variables	*τ*	*r^2^*	Parameters	a*	b*	c*
*v_0_* – MMAX - MAP	0.90	0.504	value	0.00223	−0.14530	11.77380
			*p*	0.00038	0.00807	0.00000
			error	0.00063	0.05478	1.72616
	0.50	0.431	value	0.00286	−0.32059	12.25270
			*p*	0.00000	0.00000	0.00000
			error	0.00034	0.02233	0.69262
	0.10	0.834	value	0.00270	−0.27780	8.18577
			*p*	0.00000	0.00000	0.00000
			error	0.00047	0.03881	1.24521
*v_2_* – MMAX - MAP	0.90	0.578	value	0.00039	−0.10047	6.87903
			*p*	0.00010	0.00097	0.00000
			error	3.89923	0.03040	0.92411
	0.50	0.353	value	0.00139	−0.15818	6.20199
			*p*	0.00000	0.00000	0.00000
			error	0.00024	0.02065	0.62554
	0.10	0.754	value	0.00078	−0.09694	3.52116
			*p*	0.00000	0.00000	0.00000
			error	0.00013	0.00795	0.27403
*v_∞_* – MMAX-MAP	0.90	0.551	value	0.00093	−0.06663	4.64010
			*p*	0.00135	0.00001	0.00000
			error	0.00029	0.01504	0.46712
	0.50	0.297	value	0.00075	−0.08048	3.70658
			*p*	0.00000	0.00000	0.00000
			error	0.00016	0.01216	0.36610
	0.10	0.712	value	0.00035	−0.04265	2.13045
			*p*	0.00000	0.00000	0.00000
			error	0.00007	0.00466	0.14981

Notes: * The parameters (*a*, *b*, *c)* are highly statistically significant in the models (p<0.01).

**Figure 5:**
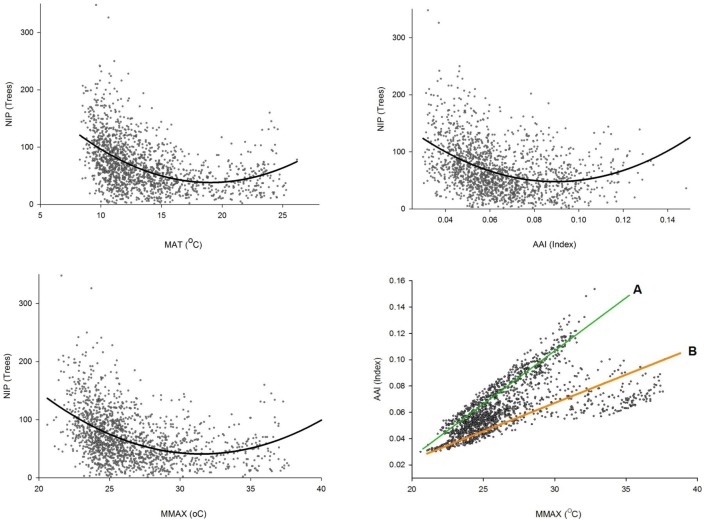
Curves of quantile regression with combinations and relationship between MMAX and AAI, Sierra Madre Occidental, Durango. Those shown are number of individuals per plot (NIP) with mean annual temperature (MAT, *r^2^* = 0.52); annual aridity index (AAI, *r^2^* = 0.47) and maximum temperature in the warmest month (MMAX, *r^2^* = 0.46). The correlation was calculated with the quadratic function by quantile regression (*τ* = 0.5). Relationship between MMAX and AAI on the Sierra Madre Occidental, Durango, A =  part facing inland and B =  part facing Pacific Ocean.

The resulting curves in Figure 5 showed that the minimum NIP occurred at the point at which the values of the most diversity-influential variables MAT, MMAX, and AAI (18.5 °C, 32.9 °C, and 0.10 days^0.5^*mm^−1^, respectively) produced almost exactly the local minimum of the tree species richness (*v_0_*) at quantile (*τ*) 0.5 (Figure 4, Table 5). In the final graph in Figure 5, the local maximum of AAI value clearly coincided with MMAX (32.9 °C), generating a local minimum of the median tree species richness (*v_0_*) at the quantile level 0.5 (Table 5).

**Figure 6:**
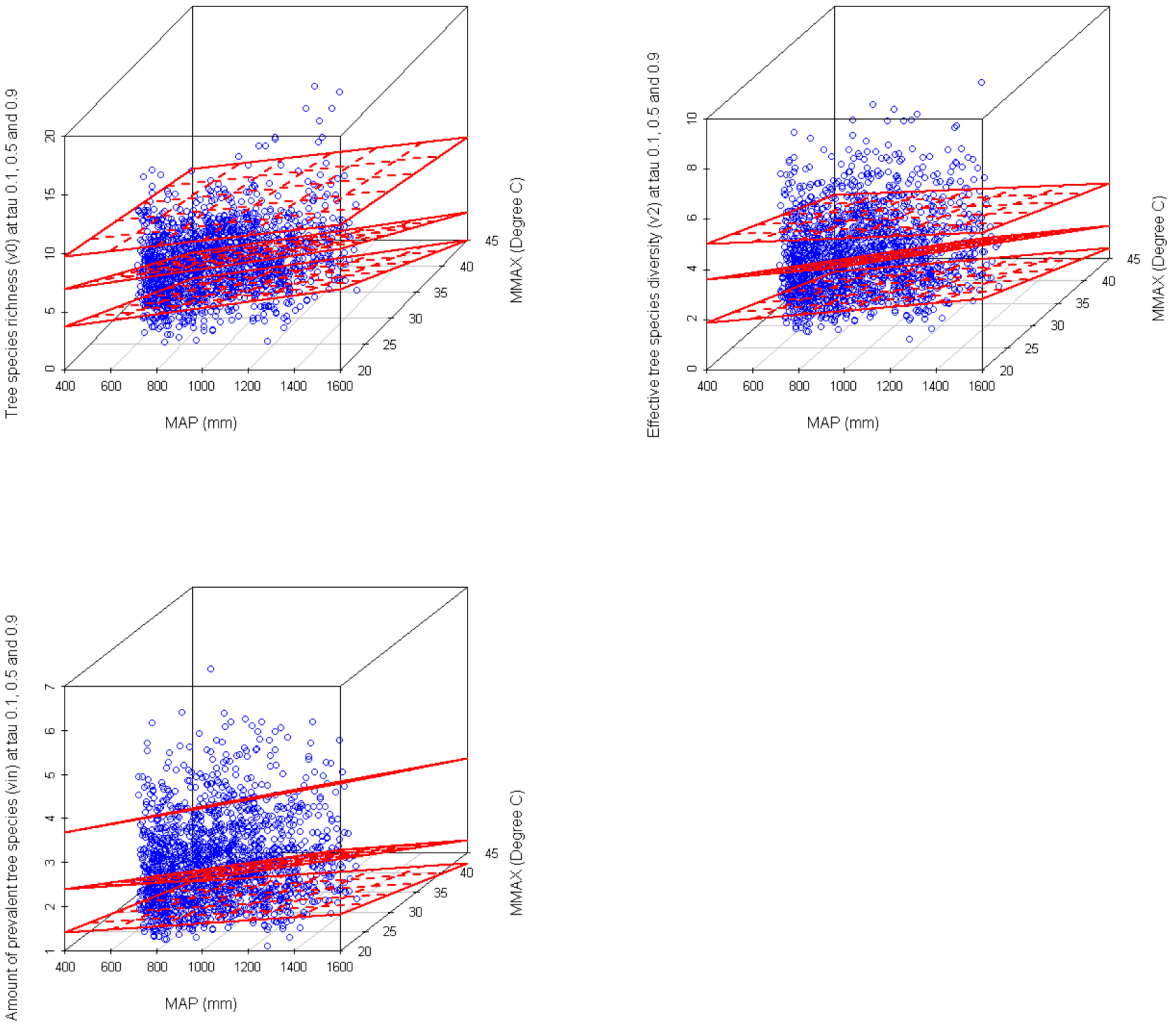
Linear multivariate model of tree species diversity with temperature and precipitation. Included tree species richness (*v_0_*), effective tree species number (*v_2_*) and the number of prevalent tree species (*v_∞_*) with mean annual precipitation (MAP) and maximum temperature in the warmest month (MMAX) for quantile levels of (*τ*) 0.1, 0.5, and 0.9.

The multivariate quantile regression of tree species diversity (*v_0_*, *v_2_* and *v_∞_* at *τ* = 0.1, 0.5 and 0.9) with MAP and MMAX showed that tree species diversity is generally higher in colder, humid temperate than in dry, hot climates in all nine models. MAP and MMAX together are good predictors of the minimum, median and maximum tree species diversity. These models show that the tree species diversity increases with MAP but decreases with MMAX (Table 6, Figure 6).

## Discussion and Conclusion

The results of this study showed that nearly all climate variables, particularly those identified in the temperature group, were weakly to moderately related to the tree species diversity in the study region, indicated using non-quantile calculations (Table 3). Similar results have been found in other studies [23], [56]. The findings also demonstrated that there is a strong relationship between the minimum, median and maximum tree species diversity and climate variables calculated by quantile regression models (Tables 4 and 6). Therefore, quantile regression could provide a more complete view of possible causal relationships between species diversity and climate variables [57].

Results support the Productivity-Diversity Hypothesis, Physiological Tolerance Hypothesis and Water-Energy Dynamic Theory, but not the Mid-Domain Effect or Metabolic Theory. The annual aridity index (AAI) was the variable most closely related to the diversity indices analyzed, although the temperature variables MAT, MTWM and MMAX were also closely associated with these variables (Table 3), as observed in other studies [21], [58], [59]. The explanation is not consistent with the Hypothesis of Evolutionary Rates and Biotic Interactions [23], or even the Metabolic Theory of Ecology [24] because diversity should be positively correlated with temperature.

However, in the present study, the relationship between diversity and temperatures in degrees Celsius was almost negative and nonlinear (Table 3, Figure 4). Hawkins et al., [34] observed significant heterogeneity in slopes among data sets, and concluded that the combined slopes across studies were significantly lower than the range of slopes predicted by metabolic theory. However, the moderate positive diversity-density relationship observed in the study (Table 3, Figure 4) confirmed that the energy-diversity relationship limits the number of individuals since climate moderately affects the net primary productivity (NPP) and thus diversity [23], [24]. The results also supported the Tolerance-Diversity Hypothesis as all diversity indices were negatively affected by the degree of aridity (Table 3, Figure 4), i.e., the harsher the climate conditions, the fewer the number of species that can maintain local conditions [58], [23]. Because of the elevation gradient, which was strongly correlated with temperature, the results also provided evidence against the Mid-Domain Effect [22] as an explanation for species diversity patterns. Furthermore, the water-energy dynamics (WED) theory [25] was also confirmed since all tree species diversities were weakly and simultaneously affected by precipitation (MAP) and temperature (MMAX) (Table 6, Figure 6). Not surprisingly, the AAI, which combined water and energy, proved to be the strongest correlate of tree diversity (Table 3). The model parameters obtained (Table 6) were highly significant for the three levels of diversity. The results obtained for the WED – tree diversity relationship calculated by linear non-quantile models was lower than the values reported by Hawkins et al., [21]. They found on average over (60%) of the variation in the richness of a wide range of plant and animal groups computed also by non-quantile statistic methods, but similarly analyzed by non-linear quantile models (Table 6). In our study, the overall weak to moderate relationship between WED and tree species richness was probably due to the small geographic scale, the species studied, and the methodology applied.

The saturation shape of the tree number per plot (NIP)-tree diversity relationship (Figure 4) may be caused by a combination of the accumulation effect [36] and increasing competition in denser plots [60], [61]. While the accumulation effect resulted in higher diversity, the self-thinning processes that took place as a result of competition in dense conditions [62] led to saturation in tree species diversity.

Interestingly, the quantile regression analysis showed that various temperature-diversity curves form a statistically significantly hollow-shaped pattern (Tables 4 and 5, Figure 4), i.e. there was a local minimum in the center of the curve. As already mentioned, this is well explained by the WED theory, which addresses the delicate balance between the temperature available to plants for growth and the available moisture (precipitation). It also provides a good explanation for the unusual regression curves because the local minimum diversity occurred almost exact when the aridity and temperature were highest and the precipitation was lowest (Figures 5 and 6). This was because lower water availability led to reduced species diversity by decreasing productivity and increasing drought stress [29], [35], [58], [63], [65].

However, the trend in the relationship between MAT and diversity was consistent with that shown in the scatter plots constructed by Hawkins et al., [34]. After transformation of the special temperature index to degrees Celsius, these scatter plots showed a local minimum diversity at a temperature of about 20°C for Californian plants, Southern African woody plants, New world ants, and of about 21–23°C for Californian and Australian butterflies and Australian amphibians.

Using non-quantile statistic methods, climatic factors had a greater effect on tree species richness (*v_0_*), in which rare tree species have greater weighting, than on the effective species number (*v_2_*) and the amount of prevalent tree species (*v_∞_*) (Table 3). The number of rare tree species, which generated the difference between values of *v_0_* and *v_2_*, was more dependent on climate than the number of more frequent species was. In contrast, the authors of a study of Scottish grassland species concluded that the richness of rare species may be intrinsically less well explained by environmental variables than the richness of common species [65]. Small populations are more exposed to genetic erosion, demographic and environmental stochasticity and natural catastrophes [66], and they are therefore at a high risk of falling below the minimum viable population size [67] and will be the first to disappear. However, in non-linear quantile regression models climatic variables had a similar effect on *v_0_*, *v_2_* and *v_∞_*, but affected the minimum, median and maximum tree species diversity differently (Tables 4 and 6).

The minimum (*τ* = 0.1), and maximum (*τ* = 0.9) diversity can be well predicted using climate variables (Tables 4 and 6). Therefore, the minimum and maximum diversity could be very sensitive indicators that can detect large-scale environmental changes (such as human-derived fragmentation and climate change) as target diversity, in contrast with the actual (observed) diversity.

Forecasts of future climate scenarios for Mexico predict an average annual temperature increase of 3.7–3.8°C, a decrease in annual precipitation of 18.2%, and an increase in aridity (AAI) of about 26% by the end of the century (in 2090) [47]. In this scenario, the diversity of tree species in the state of Durango will be drastically reduced. Assuming that these predictions are accurate, the median diversity-WED-models calculate reductions of 26% in species richness (tree species number), 25% in effective species number, and 19% in prevalent tree species in a mean plot by the year 2090. However, the model error was moderate (*r^2^* = 0.30–0.43; Table 6).

In conclusion, contemporary climate affected the minimum, median and maximum tree species diversity moderately to strongly. Water-energy dynamics provided a satisfactory explanation for the pattern of minimum, median and maximum diversity, and an understanding of this factor is therefore critical to future biodiversity research [21]. The quantile regression could be a useful tool to accurately describe the curve shape of minimum, median and maximum species diversity.
